# Zingerone suppresses angiogenesis *via* inhibition of matrix metalloproteinases during tumor development

**DOI:** 10.18632/oncotarget.10030

**Published:** 2016-06-14

**Authors:** Woom-Yee Bae, Jae-Sun Choi, Ja-Eun Kim, Chan Park, Joo-Won Jeong

**Affiliations:** ^1^ Department of Biomedical Science, Graduate School, Kyung Hee University, Seoul, 02447, Republic of Korea; ^2^ Department of Anatomy and Neurobiology, School of Medicine, Kyung Hee University, Seoul, 02447, Republic of Korea; ^3^ Department of Pharmacology, School of Medicine, Kyung Hee University, Seoul, 02447, Republic of Korea

**Keywords:** zingerone, angiogenesis, cancer, matrix metalloproteinases, JNK

## Abstract

Angiogenesis is an essential step for tumor survival and progression, and the inhibition of angiogenesis is a good strategy for tumor therapeutics. In this study, we investigated the therapeutic effect of zingerone in a mouse tumor model. Zingerone suppressed tumor progression and tumor angiogenesis. Moreover, we found that zingerone inhibited the angiogenic activities of endothelial cells by both direct and indirect means. A mechanistic study showed that the activities of MMP-2 and MMP-9 in tumor cells were decreased by treatment with zingerone. Interestingly, zingerone-mediated inhibition of MMP-2 and MMP-9 was involved in the JNK pathway. In conclusion, zingerone showed strong anti-angiogenic activity via the inhibition of MMP-2 and MMP-9 during tumor progression, suggesting that zingerone may be a potential therapeutic drug for human cancers.

## INTRODUCTION

Angiogenesis, the formation of new blood vessels, is an important process in growth and development, as well as in wound healing and the formation of granulation tissue. It comprises a complex cascade of steps, including the degradation of the basement membrane by matrix metalloproteinases (MMPs), the proliferation and migration of endothelial cells, the formation of capillaries and the stabilization of newly formed blood vessels [1, 2]. In physiological conditions, angiogenesis is tightly regulated by the production of a precise balance of activators and inhibitors. When the balance is disturbed, abnormal blood vessel growth, either excessive or insufficient, occurs in pathological conditions, including cancer, skin diseases, diabetic ulcers, cardiovascular disease, stroke, and many others. Angiogenesis is the most crucial process for providing oxygen, nutrients, and growth factors to tumor cells, especially in tumor progression [3, 4]. Many growth factors and cytokines, including vascular endothelial growth factor (VEGF), are secreted from tumor cells following exposure to hypoxic conditions, contributing to angiogenesis [5]. For some times, the blockage of angiogenesis has been accepted as an effective strategy to treat human cancer. Active research has led to the identification of angiogenic inhibitors, some of which represent therapeutic targets.

Ginger, the rhizome of *Zingiberofficinale*, is one of the most commonly used fresh herbs and spices [6]. Ginger has been used in China and India for the treatment of headache, nausea, rheumatism, cold, and diarrhea [7–9]. Ginger is typically not an allergenic food, and it is widely known to treat a number of different diseases throughout the world. A significant amount of zingerone is present in ginger (approximately 9.25%), and zingerone is known to have potent pharmacological activities. Zingerone is primarily present in dry ginger, but cooking or drying also converts gingerol into zingerone by a retroaldol reaction. Moreover, the contents of gingerol which is another component in ginger, including 6-gingerol, 8-gingerol, and 10-gingerol are commonly low in fresh ginger while on drying and roasting the amount of zingerone increases significantly. Chemically synthesized zingerone is vanillyl acetone, which is a member of the phenolic alkanone group, and its varied pharmacological properties include antioxidant, anti-inflammatory, and anticancer activities [10–16]. Natural dietary substances, such as fruits, vegetables spices, and natural pure compounds including flavonoids, and phenolic alkanones have drawn a great deal of attention from both scientists and the general public for their ability to suppress the pathogenesis of cancers. Among the natural ingredients that may be suitable for cancer treatment, ginger has a chemo-preventive effect [17]. It contains phenolic and alkanone substances that generally possess strong antioxidative and anti-inflammatory properties, as well as potent anticarcinogenic and antimutagenic activities. Although recent studies have shown that zingerone has anticancer potential [12], the antiangiogenic activity and the regulatory mechanisms of zingerone have not been defined yet.

In the present study, we demonstrated, for the first time, that zingerone significantly inhibited angiogenesis during tumor progression. We also investigated the antiangiogenic activity of zingerone in Renca cells to elucidate the underlying molecular mechanisms. Our results suggest that zingerone possesses antiangiogenic activity by inhibiting the activities of MMP-2 and MMP-9 under hypoxic conditions during tumor progression.

## RESULTS

### Zingerone inhibits tumor growth in a mouse tumor model

**Figure 1:**
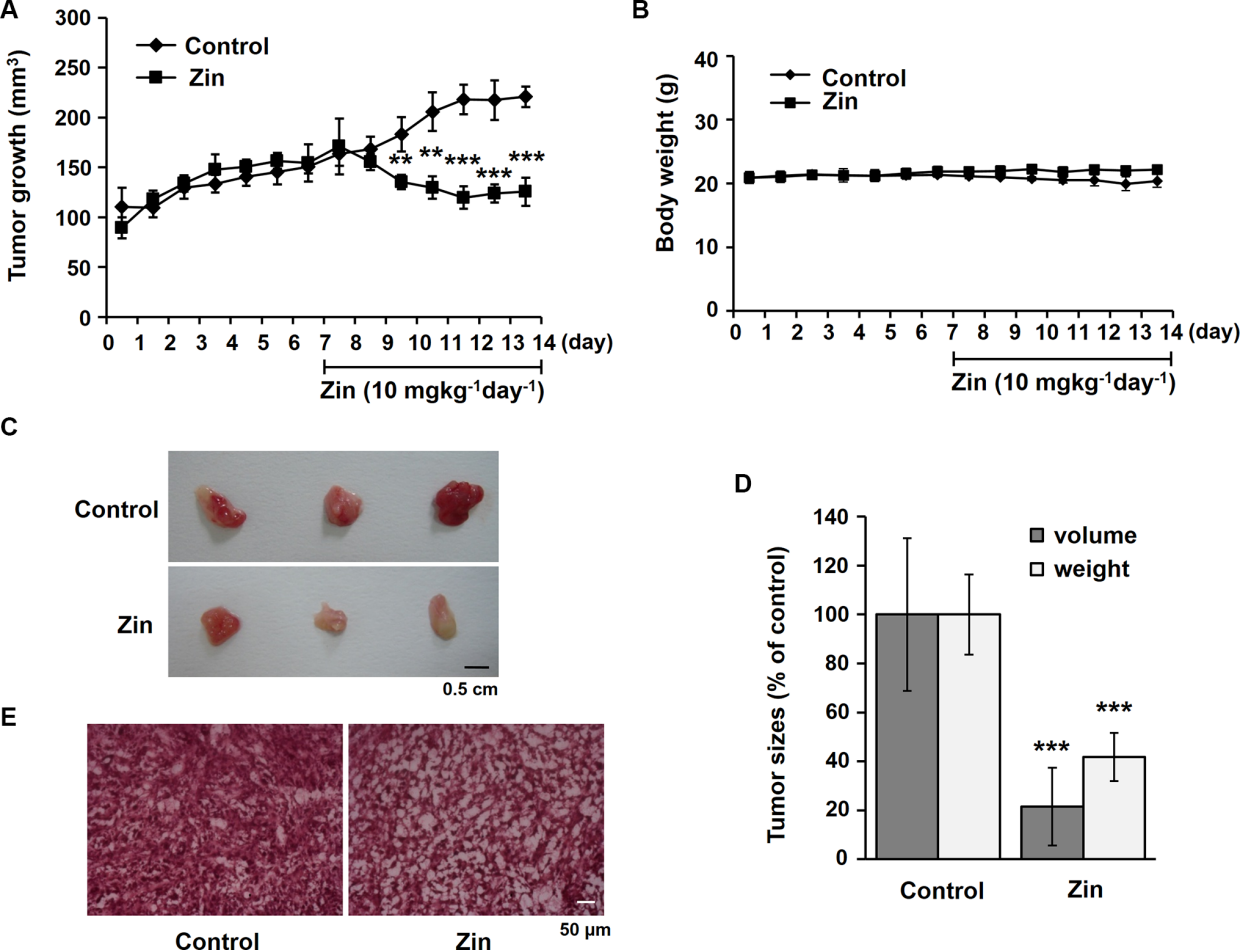
Zingerone suppresses tumor progression in a mouse tumor model (**A** and **B**) BALB/c mice were subcutaneously injected with Renca cells for a week, followed by daily treatment with zingerone for an additional week. The tumor volume and the body weight were measured using a Vernier caliper (A) and a digital balance (B), respectively, everyday for 2 weeks. The data are presented as the means ± S.D. of three independent experiments with three mice per group. **p < 0.01 and ***p < 0.001 compared to the control groups. (**C**) At day 14, the mice were sacrificed, and the tumors were isolated. The scale bar is 0.5 cm. (**D**) The tumor size and weight were measured, and the data are presented as the means ± S.D. of three independent experiments with three mice per group. ***p < 0.001 compared to the control groups. (**E**) Tumor sections were stained with hematoxylin and eosin. The scale bar is 50 μm.

To examine the effect of zingerone on tumor growth, we used a mouse tumor model stimulated by the injection of Renca cells. Gradual increases in tumor growth over a week were suppressed by the administration of zingerone for another week (Figure 1A). Moreover, the administration of zingerone did not affect the body weight of the mice (Figure 1B), suggesting that the dose of zingerone (10 mg/kg/day) used is not toxic or is at least of a low toxicity for mice. To confirm whether zingerone is not toxic, we measured the hepatic toxicity marker enzymes including glutamate oxaloacetate transaminase (GOT) and glutamate pyruvate transaminase (GPT). The mice were intraperitoneally injected with zingerone (10 mg/kg or 20 mg/kg) everyday for a week. As shown in Table 1, 10 mg/kg and 20 mg/kg of zingerone did not show any cytotoxity in mice.

**Table 1:** The effects of zingerone treatment on the activities of GOT and GPT

	Control		Zin 10 mg/kg		Zin 20 mg/kg	
	7 day	10 day	7 day	10 day	7 day	10 day
GOT(U/l)	52.7 ± 20.51	54.68 ± 17.53	40.68 ± 10.47	35.03 ± 11.3	62.37 ± 11.47	43.79 ± 9.88
GPT(U/l)	15.04 ± 1.64	11.25 ± 4.39	12.16 ± 0.83	12.68 ± 1.68	17.66 ± 3.37	15.31 ± 5.68

Values are means ± SD for three mice in each group. GOT, Glutamate oxaloacetate transaminase; GPT, Glutamate pyruvate transaminase.

Two weeks after the injection of the Renca cells, the tumor size and weight of zingerone-treated mice were significantly lower than those of the untreated control group (Figure 1C and 1D). When the cell density within the tumor tissue was examined using hematoxylin and eosin staining, we found that the tumor cell density of the zingerone-administered group was less compact than that of the control group (Figure 1E). Collectively, these results suggest that zingerone has a suppressive effect on tumor growth during tumor progression.

### Zingerone inhibits angiogenesis in tumor tissues

**Figure 2:**
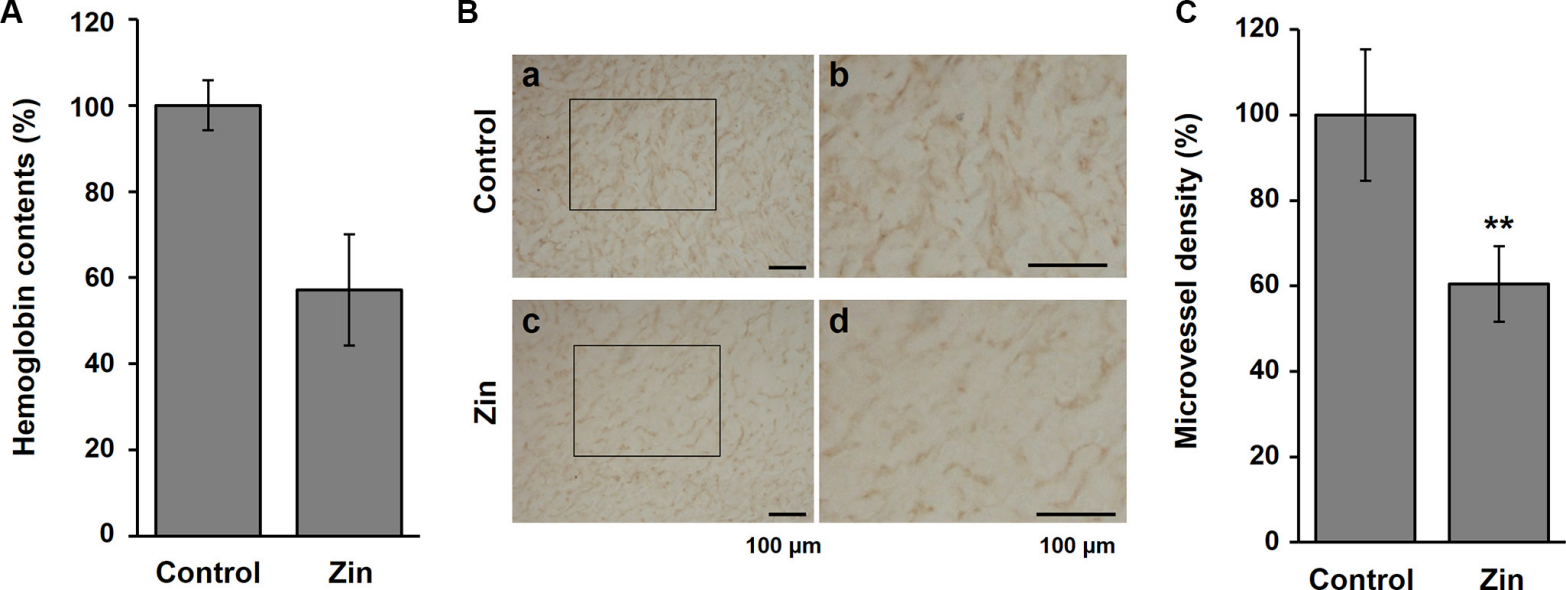
Zingerone inhibits the formation of microvessels inside tumors (**A**) The hemoglobin concentration of the isolated tumor was measured using Drabkin's reagent and was normalized to the tumor weight. (**B**) Microvessels were detected in tumor sections by immunostaining with an antibody for CD31 (1:200), a specific marker for endothelial cells. a and c, 100× magnification; b and d, 200× magnification.(**C**) The amount of CD31 was quantified using Image J software, and the data are presented as the means ± S.D. of three independent experiments. **p < 0.01 compared to the control.

Next, we wanted to determine how zingerone suppresses tumor growth. Because angiogenesis is an essential event for tumor progression, we examined the effect of zingerone on tumor angiogenesis. When we analyzed the hemoglobin contents of the tumor using Drabkin's reagent [19], we found that the concentration of hemoglobin in zingerone-administered mice was decreased (Figure 2A), suggesting that zingerone inhibits tumor angiogenesis. To explore vessel formation inside tumors, we further examined the level of CD31, a specific marker for endothelial cells, using immunohistochemistry. As shown in Figure 2B and 2C, the presence of CD31-stained capillaries in tumors was significantly attenuated by zingerone. These results suggest that zingerone could suppress tumor growth through the inhibition of tumor angiogenesis.

### Zingerone diminishes angiogenesis *in vitro*

**Figure 3:**
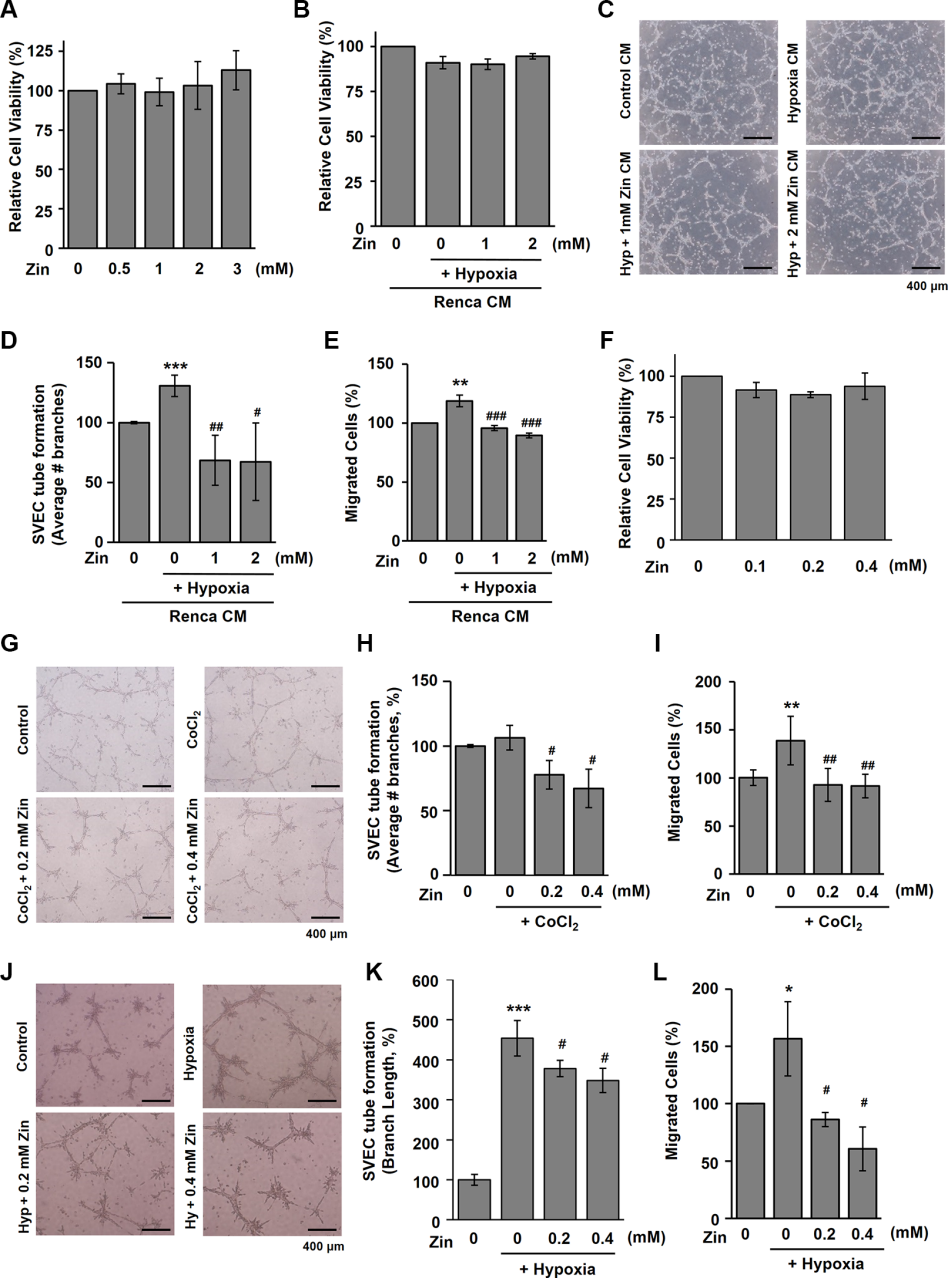
Zingerone has antiangiogenic activities *in vitro* (**A**) Renca cells were incubated with the indicated concentration of zingerone for 24 h. The data are expressed as the means ± S.D. of three independent experiments. (**B**) Under hypoxic conditions, Renca cells were incubated with or without zingerone for 24 h. CM was collected and applied to SVECs for 24 h and the data are expressed as the means ± S.D. of three independent experiments. (**C**) CM was collected and applied to SVECs in the tube formation assay. (**D**) Tube-forming area was measured using Image J software, and the data are presented as the means ± S.D. of three independent experiments. (**E**) In the wounding migration assay, migrated cells were counted, and the data are presented as the means ± S.D. of three independent experiments. **p < 0.01 and ***p < 0.001 compared to the normoxic control; ^#^*p* < 0.05, ^##^*p* < 0.01, and ^###^*p* < 0.001 compared to the hypoxic control. (**F**) SVECs were incubated with the indicated concentration of zingerone for 24 h. The data are expressed as the means ± S.D. of three independent experiments. (**G**) SVECs were incubated on Matrigel with CoCl_2_ for 10 h in the presence or absence of zingerone as indicated. (**H**) ^#^*p* < 0.05 compared to the CoCl_2_–only group. (**I**) Migrated SVECs were counted. **p < 0.01 compared to the control; ^##^*p* < 0.01 compared to the CoCl_2_-only group. (**J**) SVECs were incubated on Matrigel under hypoxia for 10 h in the presence or absence of zingerone as indicated. (**K**) ***p < 0.001 compared to the control; ^#^*p* < 0.05 compared to thehypoxia–only group. (**L**) Migrated SVECs were counted. *p< 0.05 compared to the control; ^#^*p* < 0.05 compared to the hypoxia–only group.

To assess the antiangiogenic effect of zingerone on tumor cells, *in vitro* angiogenesis assays were used. Renca cells were treated with various concentrations of zingerone for 24 h, the viability was assessed by MTT assay. Because zingerone had no significant effects on the cell viability up to 3 mM (Figure 3A), we selected 1 and 2 mM of zingerone for our further studies using Renca cells. Renca cells were treated with 1 and 2 mM of zingerone under hypoxia for 24 h, conditioned media (CM) were collected and treated into SVECs. As shown in Figure 3B, any CM did not affect the viability of SVECs. We then applied CM to SVECs in a tube formation assay and a wounding migration assay. As shown in Figure 3C and 3D, hypoxia-treated CM induced tube formation, whereas tube formation was inhibited by zingerone/hypoxia-treated CM. To confirm the antiangiogenic effect of zingerone, migration of endothelial cells was tested. As with the tube formation assay, zingerone/hypoxia-treated CM reduced migration of SVECs (Figure 3E). Furthermore, we also tested whether zingerone directly inhibits the angiogenic activity of endothelial cells. SVECs were treated with lower concentrations of zingerone for 24 h, zingerone had no significant effects on the cell viability up to 0.4 mM (Figure 3F). When zingerone was added to SVECs in addition to CoCl_2_, a hypoxia mimetic agent, as indicated, we found that the stimulated tube-forming activity and migration of SVECs by CoCl_2_ were decreased by treatment with zingerone (Figure 3G–3I). To confirm the antiangiogenic effects of zingerone, the *in vitro* assays were performed under hypoxic condition. As shown in Figure 3J–3L, stimulated tube-forming activity and migration of SVECs by hypoxic treatment were significant inhibited by zingerone treatment. From these data, we suggest that zingerone can both directly and indirectly inhibit the angiogenic activities of endothelial cells in the tumor microenvironment.

### Zingerone does not affect HIF-1α and angiogenic growth factors

**Figure 4:**
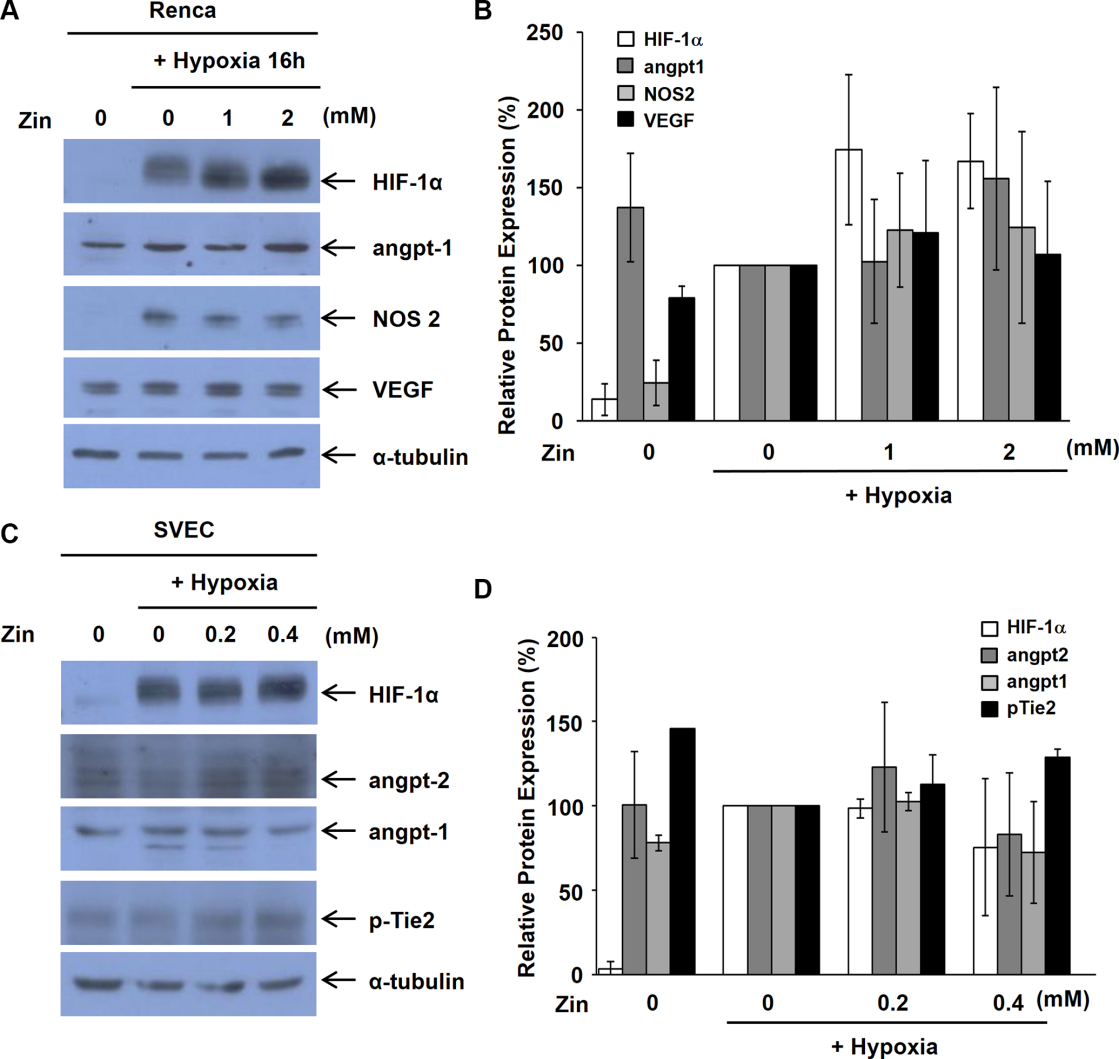
Zingerone does not inhibit the expression of HIF-1α and angiopoietins (**A**–**D**) Renca cells (A) and SVECs (C) were exposed to zingerone for 24 h and hypoxic conditions for 2 h before harvesting of whole cell extracts. Western blot analysis for HIF-1α (1:1000), VEGF (1:1000), angiopoietin-1 (1:1000), angiopoietin-2 (1:1000), Tie-2 (1:1000) and NOS2 (1:1000) was performed with the respective antibodies. α-tubulin was examined as an internal control. (B and D) Relative protein levels were expressed as the means ± S.D. of three independent experiments. The expression under hypoxic condition was set to 100%.

To identify which factors are responsible for inhibition of tumor angiogenesis by zingerone, we analyzed Western blotting of some factors involved in angiogenic mechanisms and their receptors. Although HIF-1α is a key factor for the regulation of angiogenesis during tumor progression, zingerone did not affect hypoxia-induced HIF-1α protein levels in tumor cells (Figure 4A and 4B). Moreover, VEGF, angiopoietin-1 and NOS2 protein levels were not changed by zingerone in Renca cells (Figure 4A and 4B). We also analyzed the protein levels of several factors and their receptors in endothelial cells. As shown in Figure 4C and 4D, HIF-1α, angiopoietin-1, angiopoietin-2, and angiopoietin receptor Tie-2 were not affected by zingerone treatment.

### Zingerone decreases the activities of MMP-2 and MMP-9

**Figure 5:**
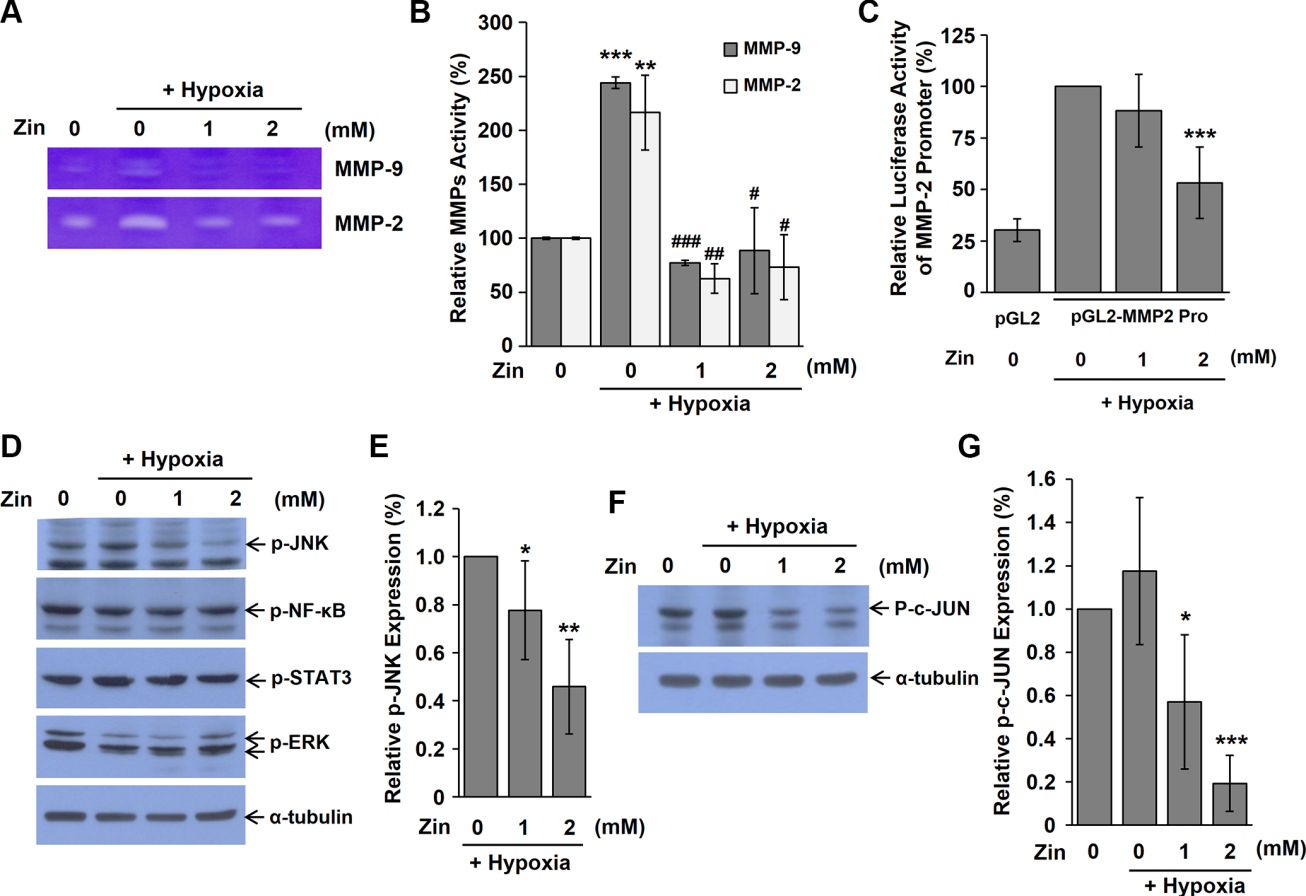
Zingerone inhibits the activities of MMP-2 and MMP-9 (**A**) CM was collected from Renca cells that were treated with zingerone for 24 h and hypoxic conditions for 2 h. CM was subjected to gelatin-based electrophoresis and the gel was stained with Coomassie brilliant blue. (**B**) The activities of MMP-2 and MMP-9 were measured by quantification of bands with Image J software. **p < 0.01 and ***p < 0.001 compared to the normoxic control; ^#^*p* < 0.05, ^##^*p* < 0.01, and ^###^*p* < 0.001 compared to the hypoxic control. (**C**) Renca cells were co-transfected with pGL2-MMP-2 promoter and pRSV-β-galatosidase vectors. After transfection, the cells were incubated in the presence of zingerone for 24 h under hypoxic conditions. Relative luciferase activity was calculated by normalization of transfection efficiency according to β-galactosidase activity. ***p < 0.001 compared to the hypoxic control. (**D**) Renca cells were exposed to zingerone for 24 h and hypoxic condition for 2 h before harvesting of whole cell extracts, and Western blot analysis was performed using indicated antibodies. p-JNK (1:1000), p-NK-κB (1:1000), p-STAT3 (1:1000), p-ERK (1:1000). (**E**) Relative phosphorylation of JNK level was expressed as the means ± S.D. of three independent experiments. The expression under hypoxic control was set to 100%. *p < 0.05, and **p < 0.01 compared to the hypoxic control. (**F**) Renca cells were exposed to zingerone for 24 h and hypoxic condition for 2 h before harvesting of whole cell extracts, and Western blot analysis was performed using p-c-Jun antibody (1:1000). (**G**) Relative phosphorylation of c-Jun level was expressed as the means ± S.D. of three independent experiments. The expression under normoxic control was set to 100%. *p < 0.05, and ***p < 0.001 compared to the hypoxic control.

Because MMPs degrade extracellular matrix (ECM) macromolecules, they are considered to play key roles in angiogenesis. To investigate whether zingerone regulates the activities of MMPs, we performed gelatin-based zymography using CM from Renca cells, as indicated. As shown in Figure 5A and 5B, the gelatinase activities of MMP-2 and MMP-9 were increased under hypoxic conditions, but zingerone treatment decreased these activities. To confirm the negative effect of zingerone on MMP-2 expression, luciferase reporter assay was performed using MMP-2 promoter vector. Under hypoxic condition, luciferase activity of MMP-2 promoter was decreased by zingerone (Figure 5C), indicating that zingerone significantly decreased expression of MMP-2. Next, to explore whether zingerone suppresses the activities of NF-κB, STAT3, ERK, and JNK, the phosphorylation of these factors was examined. Figure 5D and 5E show that zingerone treatment did not significantly affect the activation of NF-κB, STAT3 and ERK. However, treatment with zingerone significantly decreased the phosphorylation of JNK. Moreover, phospho-c-Jun that is the downstream of JNK was also decreased by zingerone treatment (Figure 5F and 5G), suggesting that JNK inactivation contributed to the reduced MMP-2 and MMP-9 expression in zingerone-treated cells.

## DISCUSSION

In this study, zingerone, a natural substance, was found to have antiangiogenic and antitumor activities in mice. Tumor growth is regulated through the orchestrated actions of apoptosis and angiogenesis within a developing tumor. In particular, angiogenesis is an essential step for the supply of nutrients and oxygen during tumor progression [20, 21]. Zingerone effectively decreased the formation of blood vessels inside developing tumors (Figure 2) and the angiogenic activities of endothelial cells (Figure 3).

The tumor microenvironment is composed of a variety of cell types that influence the angiogenic response to tumor cells. During tumor progression, hypoxia and nutrient deprivation trigger an angiogenic switch [22]. During the formation of new capillary sprouts or angiogenesis, endothelial cells digest and penetrate the underlying vascular basement membrane, invade the ECM, and form tube-like structures that continue to extend, branch and create networks, pushed by endothelial cell proliferation [23]. MMPs are known to play an important role in the degradation and remodeling of the ECM. Among several members in the MMP family, MMP-2 and MMP-9 contribute to the angiogenic process by remodeling the basement membrane to allow sprouting. The release of MMP-2 and MMP-9 from tumor cells and surrounding cells, including endothelial cells, could enhance invasion and angiogenesis during tumor progression [24]. Therefore, drugs that target MMPs can effectively inhibit tumor angiogenesis [25]. In the present study, we found that zingerone decreased the secretion of MMP-2 and MMP-9 from Renca cells (Figure 5). Angiogenic factors including VEGF can stimulate the expression and release of MMPs [26] and several signaling pathways, including NF-κB, ERK, JNK, STAT3, and p38 MAPK, and have been reported to contribute to the activation of MMP-2 and MMP-9 [27–32]. Zingerone did not inhibit the expression of these angiogenic factors (Figure 4). When we checked the activation of several signaling pathways involved in ECM remodeling, JNK was inhibited by zingerone (Figure 5). From our results, we suggest that zingerone suppressed MMPs through JNK signaling pathway, consequently inhibited tumor angiogenesis.

In previous studies, zingerone decreased the activity of NF-κB, and the protein levels of NF-κB and MMP-9 were downregulated in colitis models [33]. Therefore, zingerone might contribute to the improvement of colonic injury and the acceleration of ulcer healing in mice with colitis. Zingerone also inhibits 1,2-dimethylhydrazine-induced colon carcinogenesis in rats [16] and reduces oxidative stress as well as the activity of caspases in rats with focaltransient ischemia [34]. These collective findings suggest that zingerone could be an effective cancer therapeutic agent through antioxidant, anti-inflammatory and antiangiogenic effect on tumor and vascular endothelial cells. The novel findings presented here provide new insight into antiangiogenic and anticancer effects of zingerone and its potential use for cancer therapeutics.

## MATERIALS AND METHODS

### Materials

Zingerone and cobalt chloride (CoCl_2_) were purchased from Sigma Aldrich (St. Louis, MO, USA). Antibodies recognizing phosphorylated-p44/42 MAPK (Erk1/2)-Thr202/Tyr204, phosphorylated-Tie2 (Tyr992), phosphorylated-STAT3 (Tyr705), phosphorylated-NF-κB p65 (Ser536) and phosphorylated-SAPK/JNK (Thr183/Tyr185), phosphorylated-c-Jun (Ser63) were purchased from Cell Signaling Technology (Beverly, MA, USA). HIF-1α antibodywas obtained from NOVUS Biologicals (Littleton, CO, USA). The angiopoietin-1 and -2, Tie-2 and NOS2 antibodies were obtained from Santa Cruz (Santa Cruz, CA, USA). The CD31 antibody was obtained from BD Pharmingen (San Diego, CA, USA).

### Mouse tumor model

Male BALB/c mice (6-weeks old) were purchased from Daehan Bio-link (Chungbuk, Korea). These experiments were approved by the Committee for Care and Use of Laboratory Animals at the Kyung Hee University according to the Guide for Animal Experiments edited by the Korean Academy for Medical Sciences. The mice were maintained under a 12:12 h light/dark cycle in a temperature controlled (22 ± 1°C) room. Renca cells (5 × 10^6^), a BALB/c-derived renal adenocarcinoma cell line, were mixed 1:1 with Matrigel (BD Pharmingen, San Diego, CA, USA) and subcutaneously injected into mice. After a week, the mice were intraperitoneally (i.p.) injected with either zingerone (10 mg/kg) or saline everyday for a week. Their body weight and the tumor sizes were measured everyday for two weeks using a digital balance and a Vernier caliper, respectively. Tumor volume (mm^3^) = 0.5 [width × length × height] [18]. Two weeks after the injection of the Renca cells, the mice were sacrificed and isolated tumors were stored at −80°C until use. Four mice were used per each group. The experiments were independently repeated three times. Blood samples were drawn from the supraorbital sinus of the etherized mice and serum was collected. GOT and GPT, hepatic toxicity marker enzymes, were assessed by a commercial test kit (Asan Pharmaceutical, Seoul, Korea).

### Hemoglobin contents analysis

The hemoglobin contents from the isolated fresh tumors were measured using Drabkin's reagent (Sigma Aldrich, St. Louis, MO, USA), and the levels were normalized to the weight of tumor.

### Immunohistochemistry

The tumor tissues, which were sliced into 10 μm slices on gelatin-coated slides, were fixed with 4% paraformaldehyde for 15 min and incubated with methanol containing 0.3% hydrogen peroxide for 20 min. Then, the sections were incubated with blocking solution (1% BSA, 0.1% Triton X-100 and 1.5% FBS) at room temperature for 1 h and incubated with CD31 antibody at 4°C overnight. The tissues were washed and then incubated with biotinylated secondary antibody (Vector laboratories, Burlingame, CA, USA) and the Elite ABC Kit (Vector Laboratories, Burlingame, CA, USA). Finally, DAB solution (Sigma-Aldrich, St. Louis, MO, USA) was used to detect the immunoactivity of the tumor sections.

### Cell culture and chemical treatment

Renca cells and mouse endothelial cells (from cell line SVEC) were purchased from the American Type Culture Collection (ATCC, Rockville, MD, USA). These cells were maintained in Dulbecco's modified Eagle's medium (DMEM; WelGENE, Daegu, Korea) containing 10% FBS (Thermo Scientific, Rockford, IL, USA) and 1% penicillin / streptomycin (Cellgro, Herndon, VA, USA) in a humidified 5% CO_2_ incubator. The cells were treated with zingerone dissolved in DMSO under hypoxic condition (1% O_2_, 5% CO_2_ and 94% N_2_).

### 3-(4,5-dimethylthiazol-2-yl)-2,5-diphenyltetrazolium bromide (MTT) assay

A 0.1 mg/ml treatment of MTT (Sigma Aldrich, St Louis, MO, USA) was added to each well and incubated at 37°C for 2 h. The medium was removed from each well, and DMSO was added. The absorbance was measured at 560 nm using an iMark Microplate Absorbance Reader (Bio-Rad, Hercules, CA, USA) after dissolving. All values were expressed as the means ± standard deviation (S. D.) of three wells from three independent experiments.

### Tube formation assay

One hundred and fifty μL of Matrigel was polymerized on 24-well plates at 37°C for 30 min. SVECs (1 × 10^5^) were seeded on the surface of Matrigel and then incubated with CM containing Renca cells that either had or had not been treated with zingerone. After 10 h, morphologic changes were observed, and the branch number was counted.

### Wounding migration assay

SVECs (2 × 10^5^) were plated in 4-well plates, and the bottom of the plate was scratched using a micropipette tip. To remove the detached cells, the plate was rinsed with PBS. SVECs were treated with CM containing zingerone-treated or untreated Renca cells and allowed to migrate for 16 h. Next, the cells were fixed with absolute methanol and stained with GIEMSA (Sigma Aldrich, St. Louis, MO, USA).

### Western blot analysis

Total proteins from the indicated cells were extracted in the lysis buffer (10 mM Tris, 10 mM NaCl and 0.2% NP-40) containing a protease inhibitor cocktail (Sigma Aldrich, St. Louis, MO, USA) and phosphatase inhibitors (1 mM Na_3_VO_4_ and 10 mM NaF). For immunoblotting, total proteins were separated by SDS-PAGE and were transferred onto a PVDF membrane (Millipore Corporation, Billerica, MA, USA). The membranes were blocked with Tris-buffered saline containing 0.1% tween 20 (TBS-T) and incubated with primary antibodies diluted in blocking buffer overnight at 4°C. The membranes were washed and incubated with the appropriate HRP-conjugated secondary antibodies for 1 h at room temperature. The signal was detected using an ECL Western detection system (Thermo Scientific, Rockford, IL, USA).

### Gelatin-based zymography

Conditioned media from indicated cells were separated by SDS-PAGE using 10% acrylamide copolymerized with gelatin (0.33 mg/ml). After electrophoresis, the gel was rinsed with 2.5% Triton X-100 and incubated in the incubation buffer (0.05 M Tris, 0.15 M NaCl, 0.01 M CaCl_2_, 1 μM ZnCl_2_, 0.02% NaN_3_). Gelatinase activity was identified using Coomassie blue staining, and the digested area appeared clear on a blue background.

### Luciferase assay

Renca cells were co-transfected with pGL2-MMP2 promoter pRSV-β-galactosidase plasmids. Twenty-four hours later, the cells were incubated under hypoxic conditions for 24 h in the presence of zingerone and then lysed with luciferase cell lysis buffer. Luciferase and β-glactosidase activities were measured using luciferin and *O*-nitrophenyl-β-D-galactopyranoside, respectively. Luciferase activities were analyzed using a luminometer and the relative luciferase activity was calculated as relative light unit/β-galatosidase.

### Statistical analysis

The results are expressed as the means ± S.D. Differences between groups were examined for statistical significance using a *t*-test. A value of *p* < 0.05 was considered statistically significant.
